# Physical Activity Predicts Population-Level Age-Related Differences in Frontal White Matter

**DOI:** 10.1093/gerona/gly220

**Published:** 2020-01-20

**Authors:** Juho M. Strömmer, Simon W. Davis, Richard N. Henson, Lorraine K. Tyler, Cam-CAN, Karen L. Campbell

**Affiliations:** 1 Department of Psychology, University of Jyväskylä, Finland; 2 Department of Neurology, Duke University School of Medicine, Durham, North Carolina; 3 Medical Research Council Cognition and Brain Sciences Unit, University of Cambridge, UK; 4 Department of Psychology, University of Cambridge, UK; 5 Department of Psychology, Brock University, St Catharines, Ontario, Canada

**Keywords:** Brain aging, Exercise, Cognitive decline

## Abstract

Physical activity has positive effects on brain health and cognitive function throughout the life span. Thus far, few studies have examined the effects of physical activity on white matter microstructure and psychomotor speed within the same, population-based sample (critical if conclusions are to extend to the wider population). Here, using diffusion tensor imaging and a simple reaction time task within a relatively large population-derived sample *(N* = 399; 18–87 years) from the Cambridge Centre for Ageing and Neuroscience (Cam-CAN), we demonstrate that physical activity mediates the effect of age on white matter integrity, measured with fractional anisotropy. Higher self-reported daily physical activity was associated with greater preservation of white matter in several frontal tracts, including the genu of corpus callosum, uncinate fasciculus, external capsule, and anterior limb of the internal capsule. We also show that the age-related slowing is mediated by white matter integrity in the genu. Our findings contribute to a growing body of work, suggesting that a physically active lifestyle may protect against age-related structural disconnection and slowing.

Aging is associated with profound changes in brain structure, including gray matter atrophy and alterations in the integrity of white matter (WM). Microstructural changes in the intracellular and extracellular components of WM occur throughout the aging brain, but tend to be more pronounced in frontal associative tracts (1,2). These age-related changes are thought to be driven largely by changes in myelin, with axon fibers being relatively unaffected by age (3). Fractional anisotropy (FA), an index of microstructural WM integrity that is sensitive to changes in cerebral myelin levels, as indexed by postmortem histology (4), declines progressively with age in healthy adults, especially in those WM tracts that mature later in life, such as anterior parts of corpus callosum (5).

This loss of myelin integrity is considered one of the key mechanisms underlying normal age-related variability in cognitive performance (1,6), often surpassing gray matter volume estimates in the ability to account for age-related cognitive decline (7). Increased WM integrity is often positively correlated with better performance across a number of cognitive domains and, in some circumstances, WM integrity mediates age-related slowing of cognitive processing (8–12). In fact, a substantial portion of the age-related variance in cognitive processing speed has been shown to be attributable to decreases in frontal WM integrity (3,13). The role of WM structures like the genu of corpus callosum in mediating the effect of age on cognitive processing speed has now been replicated many times (2,12), and this relationship appears to be specific to processing speed and executive functioning, rather than other aspects of cognition (eg, language, motor functioning) (10). Thus, maintenance of WM structural connectivity appears to be particularly critical for the prevention of general age-related slowing. However, despite the ubiquity and cognitive relevance of these patterns of change in cerebral WM, the specific mediators explaining these effects—beyond chronological age itself—are unclear.

Although several lifestyle factors probably contribute to the maintenance of WM integrity with age, one of the most robust predictors of WM health appears to be physical activity. High cardiorespiratory fitness and engagement in physical activity have been shown to have protective effects for WM integrity (14–16) and cognitive performance (17–19) in healthy older adults. Evidence from prospective studies also indicates that physical activity considerably reduces the risk of dementia and Alzheimer’s disease (20). Interestingly, Burzynska and colleagues (21) showed that not only engagement in physical activity, but also avoiding sedentary behavior, is important for preserving WM microstructural integrity later in life, possibly via different pathways. Sedentary lifestyle is more likely to be associated with obesity and poor aerobic fitness, and is a leading cause of disease and disability (22), which, in turn, are shown to be associated with lower WM integrity (23). Longitudinal data from aerobic exercise intervention programs in older adults show that the selective increases in fitness associated with aerobic exercise, but not low-intensity control interventions, predict increases in WM integrity in the prefrontal and temporal cerebrum (24) and increases WM volume in the anterior corpus callosum (25). As noted earlier, these brain regions are particularly vulnerable to the detrimental effects of age. Together, these studies emphasize the potential benefits of physical activity in preventing age-related WM loss.

Although several studies suggest a link between exercise and differences in WM integrity with age (24,26), it remains to be seen whether this relationship holds within a large, population-based life span sample. Population-based samples are critical if our conclusions are to extend beyond relatively select (and potentially biased) samples of research volunteers to the population in general. Moreover, few studies, if any, have examined the relationship between brain health and participants’ reports of everyday activities and routines (encompassing such activities as cleaning the house and mode of transportation/distance to work), which arguably offer a more ecologically valid counterpoint to standard intervention studies (27). In this study, we examined the relationship between age, self-reported physical activity, WM microstructure, and processing speed within a large, population-based sample from the Cambridge Centre for Ageing and Neuroscience (Cam-CAN) (28). Participants (*N* = 399) completed a physical activity questionnaire (29) and series of cognitive tests, including simple reaction time (RT) task in their homes, before undergoing a series of structural and functional MRI scans, which included diffusion tensor imaging (28). Diffusion tensor imaging was used to estimate FA within 21 major tracts from the John Hopkins University (JHU) White Matter Atlas and related to physical activity and processing speed separately in a series of mediation models.

Our first objective was to determine whether physical activity mediates age-related decline in WM within particular tracts and whether these are the tracts that are most susceptible to age-related decline. To this end, separate mediation models were run for each tract, testing whether the relationship between age and FA was mediated by daily physical activity. Our second objective was to examine whether performance on the simple RT task is associated with WM integrity and whether the age-related decline in this measure is mediated by WM integrity. Only those tracts that showed a significant mediation effect of physical activity in the first model (corrected for multiple comparisons) were included into the second set of models testing the association between age, FA, and RT. Thus, our planned analyses will help to elucidate a possible explanation for age-related declines in WM health and provide evidence for the role of this measure in predicting declines in processing speed.

## Methods

### Participants

A healthy, population-based sample of 708 participants (age range 18–88 years) was collected as part of the Cam-CAN (for a detailed description of the study, see ref. (28)). The ethical approval for the study was obtained from the Cambridgeshire 2 (now East of England—Cambridge Central) Research Ethics Committee. Participants gave written informed consent. Exclusion criteria included poor vision (below 20/50 on Snellen test (30)), poor hearing (failing to hear 35 dB at 1,000 Hz in either ear), low Mini–Mental Status Examination (24 or lower (31)), self-reported substance abuse (assessed by the Drug Abuse Screening Test [DAST-20 (32)]), poor English knowledge (non-native or nonbilingual English speaker), current psychiatric disorder, or neurological disease. In addition, people with contraindications to MRI or MEG were excluded. Handedness was assessed using Edinburgh Handedness Inventory (33). Of the initial 708, 646 participants had valid T1, T2, and diffusion tensor imaging/diffusion kurtosis imaging data. We also excluded participants who did not complete the RT task (*n* = 75) and those with outlying FA values further than three times interquartile range above or below the age decile mean (*n* = 25; total remaining *N* = 399, 221 females, age range 18–87 years). The sample characteristics are described in Table 1.

### Imaging Preprocessing and Region-wise Analysis

The MRI data were collected from a Siemens 3T TIM TRIO (Siemens, Erlangen, Germany). To estimate WM integrity, diffusion-weighted images were acquired with a twice-refocused spin echo sequence, with 30 diffusion gradient directions each for *b* values of 1,000 and 2,000 s/mm^2^, and three images acquired using a *b* value of 0 (echo time = 104 ms, repetition time = 9.1 s, voxel size = 2 × 2 × 2 mm^3^, field of view = 192 × 192 mm^2^, 66 axial slices, GRAPPA acceleration factor = 2).

All preprocessing was completed using a combination of functions from FSL version 4.1.8 (*bet*, *eddy*, *dtifit*, and *TBSS*) and custom MATLAB scripts. The diffusion data were preprocessed for eddy currents and subject motion using an affine registration model. After removal of nonbrain tissue, a nonlinear diffusion tensor model was fit to the diffusion weighted image volumes. Nonlinear fitting of the diffusion tensor provides a more accurate noise modeling than standard linear model fitting and enables various constraints on the diffusion tensor, such as positive definiteness. The tensor’s eigensystem was used to compute the FA at each voxel; FA maps were spatially normalized into a standard stereotactic space using tract-based spatial statistics (34). Images were then smoothed with a 6 mm full width at half maximum Gaussian kernel to address possible residual errors and interindividual variability and to ensure the normality requirements of parametric statistics were met and then masked with a binarized version of each participant’s FA map, such that voxels below an FA threshold of 0.35 were not considered for further analysis.

Next, the mean FA values over 21 bilaterally symmetrical tract region of interests (ROIs) from the JHU White Matter Atlas (http://cmrm.med.jhmi.edu/) were extracted for subsequent analysis: genu of corpus callosum, body of corpus callosum, splenium of corpus callosum, column and body of fornix, fornix (cres), cerebral peduncle, anterior limb of internal capsule, posterior limb of internal capsule, retrolenticular part of internal capsule, anterior corona radiata, superior corona radiate, posterior corona radiate, posterior thalamic radiation, sagittal stratum, external capsule, cingulate gyrus, hippocampus, superior longitudinal fasciculus, superior fronto-occipital fasciculus, uncinate fasciculus, and tapetum.

### Physical Activity Questionnaire

Information about physical activity energy expenditure (PAEE) was gathered as part of a larger self-completed questionnaire, which asked about education, training, travel, hobbies, and social activities. The questions about physical activity were based on items from the European Prospective Investigation into Cancer Study–Norfolk Physical Activity Questionnaire (EPIC-EPAQ2) (29). The full questionnaire is provided in Supplementary Material. Individual total PAEE per day (kJ/d/kg) was calculated from self-reported activities into metabolic equivalents (35,36), based on the standard definition of 1 MET as 3.5 ml O_2_/min/kg (or 71 J/min/kg) based on the resting metabolic rate (37). In addition, PAEE was divided into subtypes in relation to the nature of the activity to investigate their contribution to total PAEE and age-related differences in it. Work PAEE includes all activities performed at work; Home PAEE includes home- and housework-related activities; Leisure PAEE includes all voluntary leisure activity and exercise; and Commute PAEE includes commuting to work and other travel.

### Response Time Task

In the simple RT task, participants were seated behind a computer screen and rested their right hand on a response box with four buttons (one for each finger). On the screen, they viewed an image of a hand with blank circles above each finger. Participants were instructed to press with the index finger as quickly as possible whenever the circle above the index finger in the image turned black. On pressing the button, or after maximum 3 seconds, the circle became blank again, and the variable intertrial interval began. The intertrial interval varied pseudorandomly with positively skewed distribution, minimum 1.8 seconds, mean 3.7 seconds, median 3.9 seconds, and maximum 6.8 seconds. The task included 50 trials, and mean RT was calculated for correct trials after applying a 3 *SD* trim to the data.

### Statistical Analysis

Pearson’s correlation coefficients (partialling out gender and education) were computed to examine the relationship between age and total PAEE. For the PAEE subtypes (which were skewed in their distributions), Spearman’s rank correlation coefficients were computed (partialling out gender and education) to examine age-related changes in the types of activity contributing to total PAEE.

To test whether physical activity helps to predict the effects of age-related WM decline, we ran a series of mediation analyses, in which a third mediator variable fully or partially accounts for the relationship between an independent predictor and dependent outcome variables (38). In each analysis, the independent factor was age, the dependent factor was one of the 21 WM tracts (ie, mean FA within a tract), and the mediator was the amount of physical activity (Figure 2D). For those tracks that showed a significant mediation effect, we went on to test the cognitive significance of that effect by examining the relationship between WM in those tracts and age-related slowing (Figure 2). To this end, we ran another set of mediation analyses using age as the independent factor, simple RT as the dependent factor and mean FA within each of the previously identified tracts as the mediator (Figure 2E). Direct effects of age on FA and RT were also included in these regressions. Statistical significance for mediation analyses is typically signified by a significant attenuation of the relationship (β value) between predictor and outcome variables, denoted here by a 95% confidence interval (CI) for standardized regression coefficient that does not cross zero. All significance tests were two tailed, and false discovery rate (39) at 0.05 was applied to protect against familywise Type I error.

## Results

### Aging and Physical Activity

**Figure 1:**
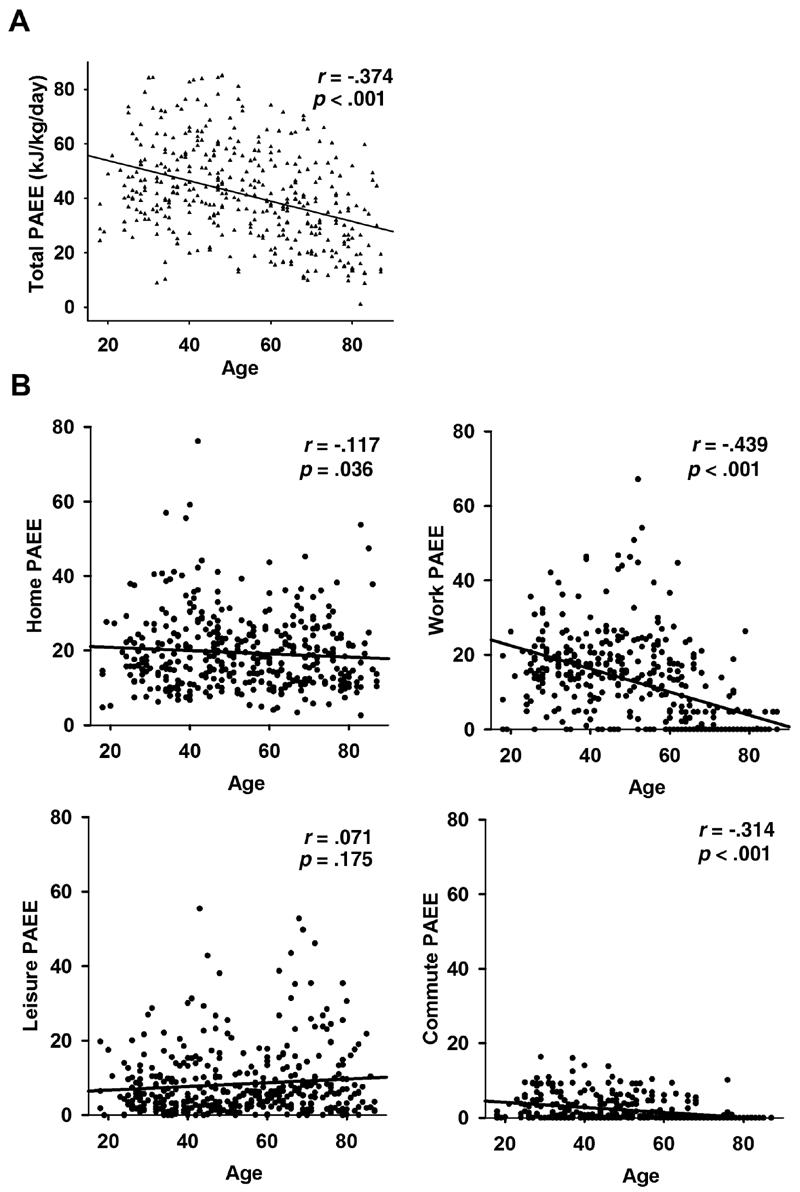
(**A**) The effect of age on total physical activity energy expenditure (PAEE). (**B**) The effect of age on PAEE subtypes of home-, work-, leisure-, and commuting-related activities.

Total PAEE, controlled for gender and education, showed a gradual decline with age, *r* = −.37, *p*_fdr_ < .001 (Figure 1A). This is also shown in the results of the mediation models as path *a*, that is, the direct negative effect of age on total PAEE (Table 2). Work-related activity (rho = −.52, *p*_fdr_ < .001) and commuting-related activity (rho = −.46, *p*_fdr_ < .001) showed moderate negative correlations with increasing age, but home-related activity showed a very weak correlation (rho = −.099, *p*_fdr_ = .06) and leisure time activity no correlation (rho = −.09, *p*_fdr_ = .09) with age (Figure 1B). To conclude, leisure- and home-related activities seem to remain stable across the life span, whereas work- and commuting-related activities decline and probably contribute to the decline in total PAEE.

### Aging and WM Integrity

**Figure 2:**
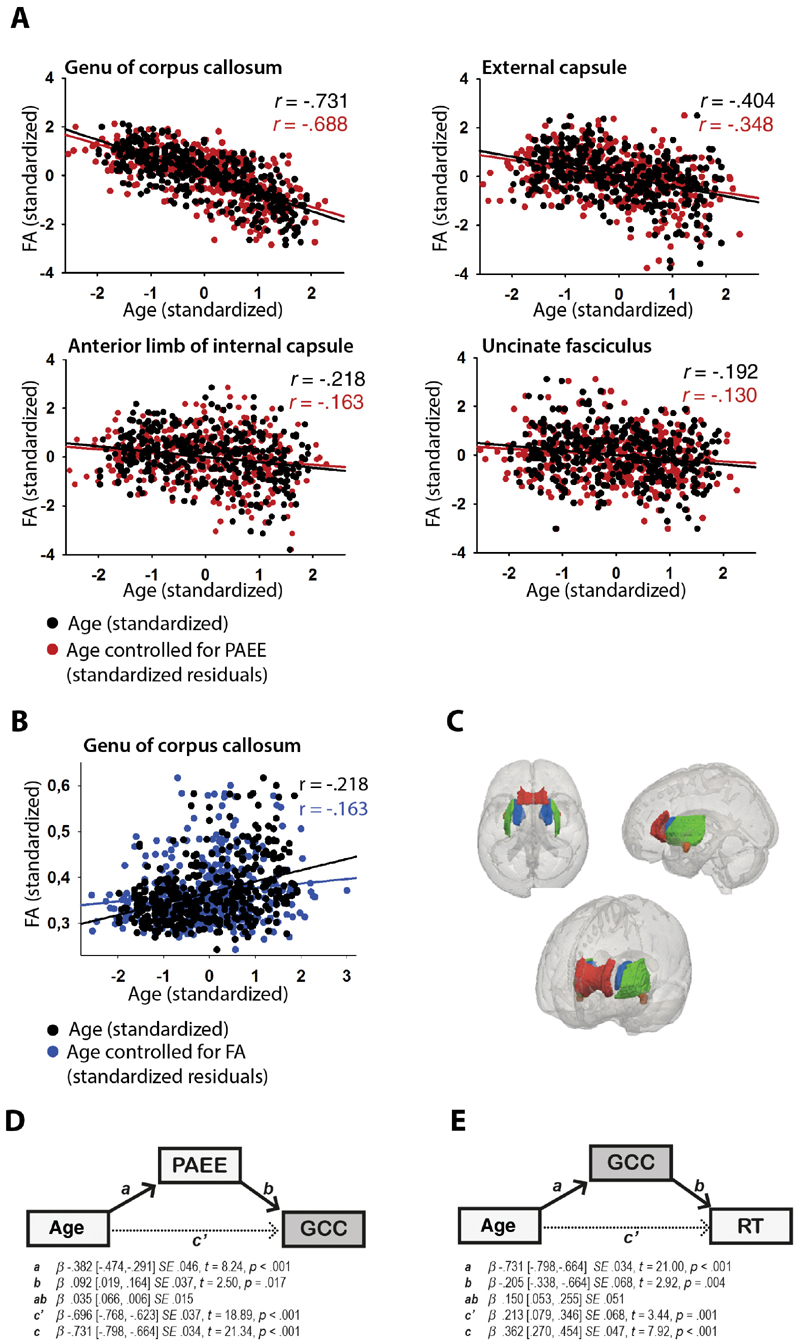
**(A)** The relationship between white matter integrity (fractional anisotropy [FA]) and age (steeper line) and age controlled for PAEE (gentlier line) in genu of corpus callosum (GCC), external capsule (EC), anterior limb of internal capsule (ALIC), and uncinated fasciculus (UNC). FA decreases gradually with age within all of the analyzed white matter tracts: GCC: *r* = −. 731, *p* < .001; EC: *r* = −. 404, *p* < .001; ALIC: *r* = −. 218, *p* < .001; UNC: *r* = −. 192, *p* < .001. The detrimental effect of age on FA is diminished in all of the analyzed tracts when PAEE is partialled out from age: GCC: *r* = −. 688, *p* < .001; EC: *r* = −. 348, *p* < .001; ALIC: *r* = −. 163, *p* = .001; UNC: *r* = −. 130, *p* = .009. The results indicate a positive relationship between higher physical activity and age-related differences in white matter microstructure. **(B)** The relationship between reaction time and age (steeper line) and age controlled for white matter integrity (FA) in genu of corpus callosum (gentlier line). Reaction times become gradually slower with age: *r* = .362, *p* < .001. The effect of age on reaction time is diminished when FA in genu of the corpus callosum is partialled out from age: *r* = .156, *p* = .002. The results indicate a positive relationship between white matter integrity in anterior corpus callosum and age-related differences in reaction time performance. **(C)** White matter tract ROIs from JHU FA atlas. Tracts which survive the first stage of mediation analysis (genu, anterior limb of the internal capsule, and the external capsule) are rendered in (left to right) superior axial, sagittal, and oblique views. **(D)** Schematic representation of the mediation paths. PAEE mediates the effect of age on FA in genu of corpus callosum. **(E)** FA in genu of corpus callosum mediates the effect of age on reaction time. Full color version is available within the online issue.

The direct effect of age on FA was negative in all of the analyzed tracts, except the posterior limb of internal capsule, which showed a small age-related increase in FA (Table 2, path *c*). The effect of age on FA was relatively large (standardized β’s < −0.5) in the genu and body of the corpus callosum, fornix, anterior corona radiata, posterior thalamic radiation, sagittal stratum, and tapetum (Figure 2A and C).

### Physical Activity and WM Integrity

The first mediation analyses tested whether total PAEE mediated the age–FA relationships. Four tracts showed a mediation effect that survived false discovery rate correction: genu of corpus callosum, anterior limb of internal capsule, external capsule, and uncinate fasciculus (Table 2, path *ab*, Figure 2A and C). The mediation effects of PAEE on these WM tracts are positive (Table 2, path *ab*), suggesting that higher physical activity is associated with less age-related WM degeneration (see Figure 2A). (These effects remain equal when controlling for gender and education, although gender has a direct effect on FA in anterior limb of internal capsule [*B* = −0.426, *SE* = 0.096, 95% CI = −0.616 to −0.237]. These analyses were also run with body mass index as a covariate to investigate the association between body mass index and PAEE. There were no associations between body mass index and PAEE, although body mass index has a marginal direct effect on external capsule [*ß* = −0.100, *SE* = 0.051, 95% CI = −0.200 to −0.001].) No mediation effects were found when different PAEE types were used as mediator instead of total PAEE.

### WM Integrity and Speed of Processing

The second mediation analyses tested whether FA (in the tracts related to exercise) mediated the relationship between age and processing speed. As expected, age was associated with slower responding on the simple RT task, *B* = 0.362, CI = 0.273 to 0.452, *SE* = 0.047 (Table 3, path *c*). Critically, mean FA in the genu of corpus callosum significantly mediated the effect of age on RT (*ab* = 0.150, CI = 0.045 to 0.251, *SE* = 0.050; Table 3, path *ab*, Figure 2B), suggesting that preservation of WM in the genu of corpus callosum is associated with less age-related slowing (Figure 2B). None of the other tracts showed significant mediation or main effects (Table 3, path *ab* and *c*). (These effects remain equal when controlling for gender and education, although gender has a direct effect on FA in anterior limb of internal capsule [*B* = -0.426, *SE* = 0.096, 95% CI = -0.616 to -0.237].)

## Discussion

This study had two major aims. First, we examined whether physical activity mediates the effects of age on WM integrity. In line with previous work, we found higher physical activity to have positive effects that may protect against the damaging effects of age on FA in anterior WM tracts, namely the genu of corpus callosum, uncinate fasciculus, anterior limb of internal capsule, and external capsule. The second aim of this study was to examine whether WM integrity within the tracts that benefit from physical activity mediates age-related slowing of processing speed. Of the four tracts tested, only the genu of the corpus callosum mediated a significant portion of the variance between age and RT on a simple motor task.

This is the first study, to our knowledge, to show a relationship between self-reported everyday activities and FA in a population-derived sample. Although our results rely on a cross-sectional sample, and thus cannot relate physical activity to rates of longitudinal change, these results suggest that those who are more physically active in their day-to-day lives also have more youth-like patterns of WM microstructure. This is consistent with previous studies focusing on healthy older individuals, which have linked higher self-reported physical activity to higher WM volume (40) and lesser WM atrophy (15). Objectively measured cardiorespiratory fitness has also been shown to be associated with FA in the cingulum (23) and large portion of the corpus callosum (14) in older adults. A recent study with two large samples of older adults demonstrated that WM tracts between prefrontal regions and medial temporal lobe are particularly associated with cardiorespiratory fitness and that these associations mediate spatial working memory performance (41). In our sample, which covers the whole adult age range from 18 to 87 years, higher everyday physical activity was associated with less age-related loss of WM in several adjacent anterior tracts. Similarly, a recent study showed that higher cardiorespiratory fitness, assessed with the maximum volume of oxygen uptake (peak VO_2_), is related to higher FA in several WM tracts in older adults (42). Their study found regional specificity in the sensitivity to cardiorespiratory fitness, including genu of corpus callosum as one of the responsive regions. As with the current results, they showed that not all WM tracts that decline with age are associated with cardiorespiratory fitness.

Overall, physical activity declined with increasing age. This appears to be due largely to a decrease in activity related to work and commuting, whereas home- and leisure-related activities remained relatively stable across the age span. These results are in line with a recent review concluding that in childhood, habituation to active lifestyle, like active travel or outdoor play, are important contributors to total daily physical activity, whereas in adulthood, life events have the greatest influence on physical activity behavior (43). In the present data, a drop in work-related activity around 60 years of age coincides with the mean retirement age in our sample. Thus, it may be that people whose everyday activity is highly dependent on the activities associated with work show the greatest drop in the total activity compared with those with an active lifestyle outside of working life. Thus, it seems particularly important to promote physical leisure activities among retired older adults, possibly with the help of societal actions.

Age-related slowing of cognitive processing has been proposed to underlie age-related declines within various domains of cognition (44). In the present study, simple RT slowed gradually with increasing age, which is a common finding among various types of age-related effects on speed of processing (45). Age-related slowing in RT was mediated by FA in the genu of corpus callosum, but not in the other tracts that related to physical activity. These findings are in line with an earlier study suggesting that WM deterioration in the anterior part of the corpus callosum may contribute to general age-related slowing (2), though other studies have also related the splenium of corpus callosum and anterior limb of internal capsule (46) and more global WM structure (12,47) to perceptual-motor speed. A recent study also showed that lower whole brain FA is linked to inefficient brain response to cognitive demands of locomotion (48).

We acknowledge that our results do not speak to causality because mediation analyses based on cross-sectional data do not inevitably represent causal relationships between age, physical activity, WM integrity, and RT. Nevertheless, we assume that age, an independent factor in both of the mediation models, cannot be changed by the influence of other factors and, furthermore, that psychomotor speed (RT) is a result of nervous system functioning (WM integrity), rather than the other way round (49). However, the causal interaction between lifestyle factors (eg, physical activity) and brain structure remains unclear: It is well known that environment and behavior, including physical activity, can cause plastic changes in the brain, but at the same time, changes in brain structure and function are known to influence behavior (ie, willingness toward action demanding physical activity). Furthermore, the strength of such inferences, based on self-reported questionnaire data, are necessarily limited. Although the reliability of such questionnaires is high (50), their absolute validity is moderate at best. Thus, observations in large samples such as ours must be validated with more time-intensive vascular measures, such as VO_2_ uptake and neuroimaging measures of cerebral perfusion. In addition, it is important to note that the Cam-CAN sample represents the population in the United Kingdom, and thus these results may not generalize to a non-Caucasian population.

To conclude, we found that self-reported levels of physical activity mediated age-related WM loss in a number of anterior tracts. Although bearing in mind the limitations of cross-sectional data and a mediation-based approach, our findings complement the evidence from previous work suggesting that a physically active lifestyle may have protective benefits against age-related structural disconnection and cognitive decline. The findings of this study further support public health recommendations about the benefits of leading a physically active lifestyle across the life span, including older adults.

## Supplementary Material

Supplementary data is available at *The Journals of Gerontology, Series A: Biological Sciences and Medical Sciences* online.

Supplementary Materials

## Figures and Tables

**Table 1:** Participant Demographic Information by Age Decile

Age Decile	1	2	3	4	5	6	7
*N* (percentage of the total 399)	28 (7)	72 (18)	70 (17)	59 (15)	67 (17)	60 (15)	43 (11)
Age range (y)	18–27	28–37	38–47	48–57	58–67	68–77	78–87
Sex (male/female)	10/18	35/37	33/37	26/33	29/38	25/35	20/23
Highest education							
University	19	64	54	40	40	29	15
A’ levels	6	4	8	10	15	13	13
GCSE grade	3	4	8	8	9	10	7
None over 16	0	0	0	1	3	8	8
MMSE	29.18 (1.0)	29.49 (1.0)	28.94 (1.2)	29.05 (1.3)	28.93 (1.3)	28.57 (1.5)	28.02 (1.4)
Simple RT_mean_ (s)	0.34 (0.04)	0.34 (0.04)	0.35 (0.06)	0.36 (0.06)	0.38 (0.06)	0.40 (0.08)	0.41 (0.07)
PAEE	43 (13)	46 (16)	52 (16)	47 (17)	38 (15)	34 (16)	25 (13)

*Note:* MMSE = Mini–Mental Status Examination; PAEE = physical activity energy expenditure (kJ/d/kg); simple RT_mean_ = mean RT on the simple RT task. Values in parentheses are *SD*.

**Table 2:** Mediation Models Testing the Mediation of the Relationship Between Age and White Matter Integrity (Fractional Anisotropy) in Genu of Corpus Callosum, Anterior Limb of Internal Capsule, External Capsule, and Uncinate Fasciculus by Physical Activity Energy Expenditure

White Matter Tract	Path *a* (Age → PAEE)				Path *b* (PAEE → FA)				Path *ab* (Mediation Effect)		Path *c’* (Residual Age → FA)				Path *c* (Age → FA)			
	*Β* (95% CI)	*BSE*	*t*	*p*	*Β* (95% CI)	*BSE*	*t*	*p*	*Β* (95% CI)	*BSE*	*Β* (95% CI)	*BSE*	*t*	*p*	*Β* (95% CI)	*BSE*	*t*	*p*
Genu of corpus callosum	−0.382 (−0.474 to −0.291)	0.046	8.24	<.001	0.092 (0.019 to 0.164)	0.037	2.50	.017	−0.035 (−0.066 to −0.006)*	0.015	−0.696 (−0.768 to −0.623)	0.037	18.89	<.001	−0.731 (−0.798 to −0.664)	0.034	21.34	<.001
Anterior limb of internal capsule	−0.382 (−0.474 to −0.291)	0.046	8.24	<.001	0.118 (0.014 to 0.221)	0.053	2.23	.033	−0.045 (−0.093 to −0.004)*	0.023	−0.173 (−0.277 to −0.070)	0.053	3.29	.001	−0.218 (−0.315 to −0.122)	0.049	4.46	<.001
External capsule	−0.382 (−0.474 to −0.291)	0.046	8.24	<.001	0.102 (0.005 to 0.200)	0.050	2.07	.049	−0.040 (−0.083 to −0.003)*	0.020	−0.365 (−0.463 to −0.268)	0.050	7.82	<.001	−0.404 (−0.495 to −0.314)	0.046	8.81	<.001
Uncinate fasciculus	−0.382 (−0.474 to -0.291)	0.046	8.24	<.001	0.141 (0.037 to 0.245)	0.053	2.66	.011	−0.054 (−0.100 to −0.013)*	0.022	−0.138 (−0.242 to −0.034)	0.053	2.61	.012	−0.192 (−0.289 to −0.096)	0.049	3.91	<.001

*Notes: Β =* standardized regression coefficient; CI = confidence interval; FA = fractional anisotropy; PAEE = Physical Activity Energy Expenditure.

* Significant mediation effects (for all effects, significance is denoted by a 95% CI that does not cross zero; false discovery rate corrected *p* value < .05).

**Table 3:** Mediation Models Testing the Mediation Between Age and Simple Reaction Time, by Correlation to the White Matter Integrity (Fractional Anisotropy) in Genu of Corpus Callosum, Anterior Limb of Internal Capsule, External Capsule, and Uncinate Fasciculus

White Matter Tract	Path *a* (Age → FA)				Path *b* (FA → RT)				Path *ab* (Mediation Effect)		Path *c’* (Residual Age → RT)				Path *c* (Age → RT)			
	*Β* (95% CI)	*BSE*	*t*	*p*	*Β* (95% CI)	*BSE*	*t*	*p*	*Β* (95% CI)	*BSE*	*Β* (95% CI)	*BSE*	*t*	*p*	*Β* (95% CI)	*BSE*	*t*	*p*
Genu of corpus callosum	−0.731 (−0.798 to −0.664)	0.034	21.00	<.001	−0.205 (−0.343 to −0.067)	0.070	2.92	.004	0.150 (0.045 to 0.251)*	0.050	0.213 (0.079 to 0.346)	0.068	3.44	.001	0.362 (0.273 to 0.452)	0.046	7.92	<.001
Anterior limb of internal capsule	−0.218 (−0.315 to −0.122)	0.049	4.46	<.001	−0.084 (−0.196 to 0.029)	0.067	1.46	.166	0.018 (−0.006 to 0.051)	0.014	0.344 (0.252 to 0.437)	0.047	7.30	<.001	0.362 (0.273 to 0.452)	0.046	7.92	<.001
External capsule	−0.404 (−0.504 to −0.305)	0.051	8.00	<.001	−0.039 (−0.150 to 0.072)	0.053	0.69	.525	0.016 (−0.023 to 0.061)	0.022	0.347 (0.247 to 0.447)	0.051	6.80	<.001	0.362 (0.273 to 0.452)	0.046	7.92	<.001
Uncinate fasciculus	−0.192 (−0.290 to −0.095)	0.050	3.87	<.001	−0.013 (−0.101 to 0.075)	0.045	0.30	.768	0.003 (−0.014 to 0.020)	0.009	0.360 (0.269 to 0.451)	0.046	7.75	<.001	0.362 (0.273 to 0.452)	0.046	7.92	<.001

*Notes: Β =* standardized regression coefficient; CI = confidence interval; FA = fractional anisotropy; RT = Reaction Time.

* Significant mediation effects (for all effects, significance is denoted by a *95%* CI that does not cross zero; false discovery rate corrected *p* value < .05).
