# Supplementary material for: Physical Activity Predicts Population-Level Age-Related Differences in Frontal White Matter
Source: J Gerontol A Biol Sci Med Sci. Author manuscript; Available in PMC 2020 Oct 21. (PMC7116228; doi:10.1093/gerona/gly220)
Supplement: Supplementary Materials [file EMS97839-supplement-Supplementary_Materials.pdf]

ID Number | | | | | | | |

PHYSICAL ACTIVITY QUESTIONNAIRE

This questionnaire is designed to find out about your physical activity in your everyday life.

Please try to answer every question, except when there is a specific request to skip a section.

Your answers will be treated as strictly confidential and will be used only for medical research

CAMB/PA/4/1201

THE QUESTIONNAIRE IS DIVIDED INTO 3 SECTIONS

- **Section A** asks about your physical activity patterns in and around the house.
- **Section B** is about travel to work and your activity at work. It may be skipped by people who have not worked at any stage during the last 12 months.
- **Section C** asks about recreations that you may have engaged in during the last 12 months.

What is your date of birth? ☐☐/☐☐/☐☐☐☐ (day / month / year)

What is today's date? ☐☐/☐☐/☐☐☐☐ (day / month / year)

Your sex *(Please tick (✓) appropriate box)*? Male ☐ Female ☐

Section A HOME ACTIVITIES

GETTING UP AND GOING TO BED

*Please put a time in **each** box*

	Average over the past year	
	At what time do you normally get up?	At what time do you normally go to bed?
On a weekday		
On a weekend day		

GETTING ABOUT — Apart from going to work

Which form of transport do you use **most often** apart from your journey to and from work?

*Please tick (✓) one box **ONLY** per line*

Distance of journeys	Usual mode of transport			
	Car	Walk	Public transport	Cycle
less than one mile				
1–5 mile(s)				
More than 5 miles				

TV OR VIDEO VIEWING

*Please put a tick (✓) on **every** line*

Hours of TV or Video watched per day	Average over the last 12 months					
	None	less than 1 hour a day	1 to 2 hours a day	2 to 3 hours a day	3 to 4 hours a day	More than 4 hours a day
On a weekday before 6 pm						
On a weekday after 6 pm						
On a weekend day before 6 pm						
On a weekend day after 6 pm						

STAIR CLIMBING AT HOME

*Please put a tick (✓) on **every** line*

Number of times you climbed up a flight of stairs (approx 10 steps) each day at home	Average over the last 12 months					
	None	1 to 5 times a day	6 to 10 times a day	11 to 15 times a day	16 to 20 times a day	More than 20 times a day
On a weekday						
On a weekend day						

ACTIVITIES IN AND AROUND THE HOME

*Please put a tick (✓) on **every** line*

Approximate number of hours each week	Average over the last 12 months						
	None	Less than 1 hour a week	1 to 3 hours a week	3 to 6 hours a week	6 to 10 hours a week	10 to 15 hours a week	More than 15 hours a week
Preparing food, cooking and washing up							
Shopping for food and groceries							
Shopping and browsing in shops for other items (e.g. clothes,toys)							
Cleaning the house							
Doing the laundry and ironing							
Caring for pre-school children or babies at home (not as paid employment)							
Caring for handicapped, elderly or disabled people at home (not as paid employment)							

Section B ACTIVITY AT WORK

Please answer this section **only** if you have been in paid employment at any time during the last 12 months or you have done regular, organised voluntary work.

If not please go to page 9

TYPES OF WORK DURING THE LAST TWELVE MONTHS

- We would like to know what full or part-time jobs you have done in the last 12 months.
- You may have held a single job or have held two jobs at once.
- If you have changed jobs with the same employer, you should enter it as a change of job **only** if it entailed a substantial change in physical effort.

EXAMPLE

Someone who worked full-time for 6 months, then retired, rested for 3 months and then started a voluntary job for 6 hours a week, would complete the questions as follows.

	Job 1	Job 2
Name of occupation	*nurse*	*shop work*
How many hours **per week** did you usually work?	*38*	*6*
For how many months in the last 12 months did you do this work?	*6*	*3*

ACTIVITY LEVELS AT YOUR WORK

Now we would like you to take the total number of hours you worked per week in each job and divide them up according to your activity level.

Please complete EACH line

	Job 1			Job 2		
	No	Yes	Hours per week	No	Yes	Hours per week
Sitting — light work<br>e.g. desk work, or driving a car or truck		✓	*6*	✓		
Sitting — moderate work<br>e.g. working heavy levers or riding a mower or forklift truck	✓				✓	*2*
Standing — light work<br>e.g. lab technician work or working at a shop counter		✓	*30*		✓	*4*
Standing — light/moderate work<br>e.g. light welding or stocking shelves		✓	*2*	✓		

The number of hours in each activity should add up to the number of hours that you worked in each job e.g. 6+30+2=38 (nurse)

What jobs have you held in the last 12 months, and how many months in the year did you do them?

Please complete EACH line

	Job 1	Job 2
Name of occupation		
How many hours **per week** did you usually work?		
For how many months in the last 12 months did you do this work?		

ACTIVITY LEVELS AT YOUR WORK

Now we would like you to take the total number of hours you worked per week in each job and divide them up according to your activity level.

Please complete EACH line

	Job 1			Job 2		
	No	Yes	Hours per week	No	Yes	Hours per week
Sitting — light work<br>e.g. desk work, or driving a car or truck						
Sitting — moderate work<br>e.g. working heavy levers or riding a mower or forklift truck						
Standing — light work<br>e.g. lab technician work or working at a shop counter						
Standing — light/moderate work<br>e.g. light welding or stocking shelves						
Standing — moderate work<br>e.g. fast rate assembly line work or lifting up to 50 lbs every 5 minutes for a few seconds at a time						
Standing — moderate/heavy work<br>e.g. masonry/painting or lifting more than 50 lbs every 5 minutes for a few seconds at a time						
Walking at work — carrying nothing heavier than a briefcase<br>e.g. moving about a shop						
Walking — carrying something heavy						
Moving, pushing heavy objects objects weighing over 75lbs						

STAIR OR STEP CLIMBING AT WORK

Please put a tick (✓) on EACH line where appropriate

Number of times you climbed up a flight of stairs (10 steps) at work	AVERAGE OVER THE LAST 12 MONTHS					
	None	1 to 5 times a day	6 to 10 times a day	11 to 15 times a day	16 to 20 times a day	More than 20 times a day
Job 1						
Job 2						

Please put a tick (✓) on EACH line where appropriate

Number of times you climbed up a ladder at work	AVERAGE OVER THE LAST 12 MONTHS					
	None	1 to 5 times a day	6 to 10 times a day	11 to 15 times a day	16 to 20 times a day	More than 20 times a day
Job 1						
Job 2						

KNEELING AND SQUATTING AT WORK IN JOB 1

In an average working day in Job 1 did you

kneel for more than one hour in total? No ☐ Yes ☐ Don't know ☐

squat for more than one hour in total? No ☐ Yes ☐ Don't know ☐

get up from kneeling or squatting more than 30 times? No ☐ Yes ☐ Don't know ☐

KNEELING AND SQUATTING AT WORK IN JOB 2

In an average working day in Job 2 did you

kneel for more than one hour in total? No ☐ Yes ☐ Don't know ☐

squat for more than one hour in total? No ☐ Yes ☐ Don't know ☐

get up from kneeling or squatting more than 30 times? No ☐ Yes ☐ Don't know ☐

TRAVEL TO AND FROM WORK

JOB 1

Please complete EVERY line

Roughly how many miles was it from home to Job 1?	
How many times a week did you travel from home to Job 1?	

Please tick (✓) one box ONLY per line

**How did you normally travel to Job 1?**	Always	Usually	Occasionally	Never or rarely
By car				
By works or public transport				
By bicycle				
Walking				

JOB 2 *(if appropriate)*

Please complete EVERY line

Roughly how many miles was it from home to Job 2?	
How many times a week did you travel from home to Job 2?	

Please tick (✓) one box ONLY per line

**How did you normally travel to Job 2?**	Always	Usually	Occasionally	Never or rarely
By car				
By works or public transport				
By bicycle				
Walking				

Section C RECREATION

The following questions ask about how you spent your leisure time.

Please indicate how often you did each activity on average over the last 12 months.

For activities that are seasonal, e.g. cricket or mowing the lawn, please put the average frequency during the season when you did the activity.

Please indicate the average length of time that you spent doing the activity on each occasion.

EXAMPLE

If you had mowed the lawn every fortnight in the grass cutting season and took 1 hour and 10 minutes on each occasion.

If you went walking for pleasure for 40 minutes once a week.

You would complete the table below as follows:

Please give an answer for the AVERAGE TIME you spent on each activity and the NUMBER OF TIMES you did that activity in the past year.

	Number of times you did the activity in the last 12 months								Average time per episode	
	None	Less than once a month	Once a month	2 to 3 times a month	Once a week	2 to 3 times a week	4 to 5 times a week	Every day	Hours	Mins
Mowing the lawn				✓					1	10
Walking for pleasure					✓					40

Now please complete the table on pages 10 and 11

Please give an answer for the NUMBER OF TIMES you did the following activities in the last 12 months and the AVERAGE TIME you spent on each activity.

Please complete EACH line

	Number of times you did the activity in the last 12 months								Average time per episode	
	None	Less than once a month	Once a month	2 to 3 times a month	Once a week	2 to 3 times a week	4 to 5 times a week	6 times a week or more	Hours	Mins
Swimming — competitive										
Swimming — leisurely										
Backpacking or mountain climbing										
Walking for pleasure — you should not include walking as a means of transportation as this was included in Sections A & B										
Racing or rough terrain cycling										
Cycling for pleasure — you should not include cycling as a means of transportation										
Mowing the lawn — during the grass cutting season										
Watering the lawn or garden in the summer										
Digging, shovelling or chopping wood										
Weeding or pruning										
DIY e.g. carpentry, home or car maintenance										
High impact aerobics or step aerobics										
Other types of aerobics										
Exercises with weights										
Conditioning exercises e.g. using an exercise bike or rowing machine										

Please continue on the next page

Please complete EACH line

	Number of times you did the activity in the last 12 months								Average time per episode	
	None	Less than once a month	Once a month	2 to 3 times a month	Once a week	2 to 3 times a week	4 to 5 times a week	6 times a week or more	Hours	Mins
Floor exercises e.g. stretching, bending, keep fit or yoga										
Dancing e.g. ballroom or disco										
Competitive running										
Jogging										
Bowling — indoor, lawn or 10 pin										
Tennis or badminton										
Squash										
Table tennis										
Golf										
Football, rugby or hockey (during the season)										
Cricket (during the season)										
Rowing										
Netball, volleyball or basketball										
Fishing										
Horse-riding										
Snooker, billiards or darts										
Musical instrument playing or singing										
Ice-skating										
Sailing, wind-surfing or boating										
Martial arts, boxing or wrestling										

You have finished the questionnaire — *Thank you*

Produced by: MeDesign (01865 426219) Ref: CAMB/PA/4/1201

Cam-CAN Self completion questionnaire

The Cambridge Centre for Ageing and Neuroscience Study (Cam-CAN)

Understanding healthy ageing: Lifespan development of brain and mind

Cam-CAN ID Number:

Date questionnaire completed:

This Cam-CAN paper questionnaire is intended to capture aspects of your life that are difficult to measure during the face-to face interview. It consists of two areas:

i) a lifetime experiences questionnaire
ii) a questionnaire on physical activity.

Please complete the questionnaire before your home interview; if you have any questions feel free to ring for advice.

To complete the questionnaire, please continue and answer each question as accurately as possible. Completing the questionnaire will take about 20-40 minutes. The Cam-CAN research interviewer will collect the questionnaire during the home interview.

Thank you for your participation.

LIFETIME EXPERIENCE QUESTIONNAIRE

This questionnaire covers your current activities, your training courses and your employment history. Please complete all sections relevant to you. You may be asked to skip sections.

YOUNG ADULTHOOD TRAINING

Answer for your life experiences to date for all types of training or study.

**Type of Course**	**Number of years completed**	**Full or part time**
CSE		
NVQ level 1 / BTEC Introductory		
O level / GCSE / leaving certificate		
NVQ level 2 / BTEC First diploma		
A level / International baccalaureate		
NVQ level 3 / BTEC Diploma		
HNC / HND / NVQ level 4 / BTEC Professional		
BTEC Advanced		
College diploma		
University Undergraduate		
University Masters		
University PhD / Doctorate		
Clerical, administrative or book-keeping course		
Business course		
Trade apprenticeship		
Other professional course<br>(specify)		
Other technical course<br>(specify)		
Other graduate course<br>(specify)		
Any other course<br>(specify)		

YOUNG ADULTHOOD ACTIVITIES

Have you ever travelled to any of the following places since the age of 13?
Please tick (✓) all that apply

- ○ Around the UK/Ireland away from where I lived
- ○ Northern Europe / Scandinavia (e.g. France, Germany, Norway, Greenland)
- ○ Southern Europe (e.g. Italy, Spain)
- ○ Eastern Europe (e.g. Russia, Romania)
- ○ Northern Africa / Middle East (e.g. Tunisia, Egypt)
- ○ Southern Africa / Asia (e.g. Kenya, Madagascar, Mauritius, China, India)
- ○ North America (e.g. USA, Canada including Alaska, Hawaii)
- ○ South America / Central America / Caribbean (e.g. Mexico, Cuba, Brazil)
- ○ Australia / New Zealand / Pacific Islands / Antarctica (e.g. Fiji, Togo)
- ○ None of the above

From the ages of 13 to date please indicate how often you ever have done the following activities (please tick ✓ box)

**Type of activity**	**Never**	**Less than once a month**	**Once a month**	**Every two weeks**	**Every week**	**Daily**
Make an outing to see a family member, friend or group of friends						
Practise or play a musical instrument						
Practise or develop an artistic pastime (e.g. drawing, acting, writing etc.)						
Mildly energetic activities (e.g. walking, carpentry, gardening, housework)						
Moderately energetic activities (e.g. dancing, golf, lawn mowing, easy cycling)						
Vigorous energetic activities (e.g. running, squash, competitive tennis)						
Read (material or any sort)						
Speak a second language						
Computer games / games consoles						
Social networking / internet surfing						
Crossword puzzles / sudoku						
Strategic games (e.g. chess)						
Prayer / religious activities						

RECENT ACTIVITIES

What activities do you currently undertake during a typical week?
Please tick (✓) all that apply

- ○ Socialising (face-to-face)
- ○ Reading
- ○ Writing
- ○ Studying
- ○ Teaching
- ○ Volunteer work
- ○ Paid work
- ○ Social networking sites
- ○ Twitter / texting
- ○ None of the above
- ○ Prayer / religious activity
- ○ Helping friends / family
- ○ Artistry
- ○ Strategic games (e.g. chess)
- ○ Learning something new
- ○ Hobby pastime
- ○ Brain training games
- ○ Other computer based games
- ○ Crossword puzzles / sudoku

What type of events or entertainment have you undertaken in the **last two months**? Please tick (✓) all that apply

- ○ Going to the cinema
- ○ Going to the pub
- ○ Going to pop concerts
- ○ Going to sporting events
- ○ None of the above
- ○ Going to the theatre
- ○ Going to a restaurant
- ○ Going to festivals
- ○ Going to clubs / societies

How do you usually acquire information about world and national events?
Please tick (✓) all that apply

- ○ No particular way
- ○ TV
- ○ Newspapers
- ○ Internet
- ○ Friends / word of mouth
- ○ Radio
- ○ Magazines
- ○ Other (please give details)

What kinds of materials are you reading on a regular basis?
Please tick (✓) all that apply

- ○ Newspaper articles
- ○ Novel / books
- ○ Other (please give details)
- ○ Magazine articles
- ○ Internet blogs / articles
- ○ None of the above

Have you travelled to any of the following places in the last **five** years?
Please tick (✓) all that apply

- ○ Around the UK/Ireland away from where I lived
- ○ Southern Europe (e.g. Italy, Spain)
- ○ Northern Africa / Middle East (e.g. Tunisia, Egypt)
- ○ North America (e.g. USA, Canada including Alaska, Hawaii)
- ○ Australia / New Zealand / Pacific Islands / Antarctica (e.g. Fiji, Togo)
- ○ Northern Europe / Scandinavia (e.g. France, Germany, Norway, Greenland)
- ○ Eastern Europe (e.g. Russia, Romania)
- ○ Southern Africa / Asia (e.g. Kenya, Madagascar, Mauritius, China, India)
- ○ South America / Central America / Caribbean (e.g. Mexico, Cuba, Brazil)
- ○ None of the above

BLANK PAGE

EMPLOYMENT

You will be asked about the **main jobs** that you have undertaken for different periods of your life. The enclosed guide gives examples of job titles and how we will code them. Please enter this sort of title. We also ask for the number of people you employed, or supervised during that period.

Age	SOC number (1-10) or JOB TITLE	Number of people in charge of
18-19	Not employed	
20-24	Shop assistant	0
25-29	Secretary	0
30-34	Ages not applicable	
35-39	Ages not applicable	
40-44	Ages not applicable	
45-49	Ages not applicable	
50-54	Ages not applicable	
55-59	Ages not applicable	
60-64	Ages not applicable	
65-69	Ages not applicable	
70-74	Ages not applicable	
75 years and above	Ages not applicable	

Example

Please enter the job titles below, please try and be as explicit as possible. If you were in charge of people please indicate the approximate number. If you are not sure of the code enter your job title in the box provided.

**Age**	**SOC number (1-10) or JOB TITLE**	**Number of people in charge of**
18-19		
20-24		
25-29		
30-34	Ages not applicable	
35-39	Ages not applicable	
40-44	Ages not applicable	
45-49	Ages not applicable	
50-54	Ages not applicable	
55-59	Ages not applicable	
60-64	Ages not applicable	
65-69	Ages not applicable	
70-74	Ages not applicable	
75 years and above	Ages not applicable	

BLANK PAGE

PHYSICAL ACTIVITY

This questionnaire is designed to find out about your physical activity in your everyday life.

Please try to answer every question, except when there is a specific request to skip a section.

THE QUESTIONNAIRE IS DIVIDED INTO 3 SECTIONS

Section A asks about your physical activity patterns in and around the house.
Section B is about travel to work and your activity at work.
It may be skipped by people who have not worked at any stage during the last 12 months.
Section C asks about recreations in which you may have engaged during the last 12 months.

Section A HOME ACTIVITIES

GETTING UP AND GOING TO BED

Please put a time in **each** box

	Average over the past year	
	At what time do you normally get up?	At what time do you normally go to bed?
On a weekday		
On a weekend day		

GETTING ABOUT – Apart from going to work

Which form of transport do you use **most often** apart from your journey to and from work?

Please tick (✓) one box **ONLY** per line

Distance of journeys	Usual mode of transport			
	Car	Walk	Public transport	Cycle
Less than one mile				
1–5 mile(s)				
More than 5 miles				

TV OR VIDEO VIEWING

Please put a tick (✓) on **EVERY** line

Hours of TV or Video watched per day	Average over the last 12 months					
	None	Less than 1	1 to 2	2 to 3	3 to 4	More than 4
On a weekday before 6 pm						
On a weekday after 6 pm						
On a weekend day before 6 pm						
On a weekend day after 6 pm						

STAIR CLIMBING AT HOME

Please put a tick (✓) on **EVERY** line

Number of times you climbed up a flight of stairs (approx 10 steps) each day at home	**Average over the last 12 months**					
	None	1 to 5	6 to 10	11 to 15	16 to 20	More than 20
On a weekday						
On a weekend day						

ACTIVITIES IN AND AROUND THE HOME

Please put a tick (✓) on **EVERY** line

Approximate number of hours each week	**Average hours per week over the last 12 months**						
	0	Less than 1	1 to 3	3 to 6	6 to 10	10 to 15	>15
Preparing food, cooking and washing up							
Shopping for food and groceries							
Shopping and browsing in shops for other items (e.g. clothes, toys)							
Cleaning the house							
Doing the laundry and ironing							
Caring for pre-school children or babies at home (not as paid employment)							
Caring for handicapped, elderly or disabled people at home (not as paid employment)							

Section B ACTIVITY AT WORK

Please answer this section **only** if you have been in paid employment at any time during the last 12 months or you have done regular, organised voluntary work.

IF YOU HAVE NOT BEEN EMPLOYED DURING THE LAST 12 MONTHS PLEASE GO TO PAGE 16

TYPES OF WORK DURING THE LAST TWELVE MONTHS

- We would like to know what full or part-time jobs you have done in the last 12 months.
- You may have held a single job or have held two jobs at once.
- If you have changed jobs with the same employer, you should enter it as a change of job **only** if it entailed a substantial change in physical effort.

EXAMPLE

Someone who worked full-time for 6 months, then retired, rested for 3 months and then started a voluntary job for 6 hours a week, would complete the questions as follows

	**Job 1**	**Job 2**
Name of occupation	nurse	shop work
How many hours **per week** did you usually work?	38	6
For how many months in the last 12 months did you do this work?	[illegible]	3

Example

ACTIVITY LEVELS AT YOUR WORK

Please complete EACH line

	**Job 1**			**Job 2**		
	No	Yes	Hours per week	No	Yes	Hours per week
Sitting — light work<br>e.g. desk work, or driving a car or truck		✓	6	✓		
Sitting — moderate work<br>e.g. working heavy levers or riding a mower or forklift truck	✓				✓	2
Standing — light work<br>e.g. lab technician work or working at a shop counter		✓	30		✓	4
Standing — light/moderate work<br>e.g. light welding or stocking shelves		✓	2	✓		

Example

The number of hours in each activity should add up to the number of hours that you worked in each job e.g. 6+30+2=38 (nurse)

What jobs have you held in the last 12 months, and how many months in the year did you do them?

Please complete EACH line

	**Job 1**	**Job 2**
Name of occupation		
How many hours **per week** did you usually work?		
For how many months in the last 12 months did you do this work?		

ACTIVITY LEVELS AT YOUR WORK

Now we would like you to take the total number of hours you worked per week in each job and divide them up according to your activity level.

Please complete **EACH** line

	**Job 1**			**Job 2**		
	No	Yes	Hours per week	No	Yes	Hours per week
Sitting — light work e.g. desk work, or driving a car or truck						
Sitting — moderate work e.g. working heavy levers or riding a mower or forklift truck						
Standing — light work e.g. lab technician work or working at a shop counter						
Standing — light/moderate work e.g. light welding or stocking shelves						
Standing — moderate work e.g. fast rate assembly line work or lifting up to 50 lbs (25 kg) every 5 minutes for a few seconds at a time						
Standing — moderate/heavy work e.g. masonry/painting or lifting more than 50 lbs (25 kg) every 5 minutes for a few seconds at a time						
Walking at work — carrying nothing heavier than a briefcase e.g. moving about a shop						
Walking — carrying something heavy						
Moving, pushing heavy objects weighing over 75lbs (35kg)						

STAIR OR STEP CLIMBING AT WORK
Please put a tick (✓) on EACH line where appropriate

Number of times a day you climbed up a flight of stairs (10 steps) at work	**AVERAGE NUMBER OF TIMES A DAY OVER THE LAST 12 MONTHS**					
	0	1 to 5	6 to 10	11 to 15	16 to 20	More than 20
Job 1						
Job 2						

Number of times a day you climbed up a ladder at work	**AVERAGE NUMBER OF TIMES A DAY OVER THE LAST 12 MONTHS**					
	0	1 to 5	6 to 10	11 to 15	16 to 20	More than 20
Job 1						
Job 2						

In an average working day in **job 1** did you
(please tick ✓ all that apply)

- ○ Kneel for more than an hour in total?
- ○ Squat for more than an hour in total?
- ○ Get up from kneeling or squatting more than 30 times?

In an average working day in **job 2** did you

- ○ Kneel for more than an hour in total?
- ○ Squat for more than an hour in total?
- ○ Get up from kneeling or squatting more than 30 times?

TRAVEL TO AND FROM WORK

JOB 1

Please complete **EVERY** line

Roughly how many miles was it from home to Job 1?	
How many times a week did you travel from home to Job 1?	

Please tick (✓) one box **ONLY** per line

How did you normally travel to Job 1?	Always	Usually	Occasionally	Never or rarely
By car				
By works or public transport				
By bicycle				
Walking				

JOB 2 *(if appropriate)*

Please complete **EVERY** line

Roughly how many miles was it from home to Job 2	
How many times a week did you travel from home to Job 2	

Please tick (✓) one box **ONLY** per line

How did you normally travel to Job 2?	Always	Usually	Occasionally	Never or rarely
By car				
By works or public transport				
By bicycle				
Walking				

Section C RECREATION

The following questions ask about how you spent your leisure time. Please indicate how often you did each activity on average over the last 12 months. For activities that are seasonal, e.g. cricket or mowing the lawn, please put the average frequency during the season when you did the activity. Please indicate the average length of time that you spent doing the activity on each occasion.

EXAMPLE
If you had mowed the lawn every fortnight in the grass cutting season and took 1 hour and 10 minutes on each occasion, and you went walking for pleasure for 40 minutes once a week, you would complete the table below as follows:

Please give an answer for the AVERAGE TIME you spent on each activity and the NUMBER OF TIMES you did that activity in the past year.

	Number of times you did the activity in the last 12 months								Average time per episode	
	None	Less than once a month	Once a month	2 to 3 times a month	Once a week	2 to 3 times a week	4 to 5 times a week	Daily	Hours	Mins
Mowing the lawn				✓					1	10
Walking for pleasure					✓					40

For activities you do as a means of transportation, please do not include those in this table.

Now please complete the table on pages 17-18

Please give an answer for the NUMBER OF TIMES you did the following activities in the last 12 months and the AVERAGE TIME you spent on each activity.

Please complete EACH line

	Number of times you did the activity in the last 12 months								Average time per episode	
	None	Less than once a month	Once a month	2 to 3 times a month	Once a week	2 to 3 times a week	4 to 5 times a week	Daily	Hours	Mins
Swimming — competitive										
Swimming — leisurely										
Backpacking or mountain climbing										
Walking for pleasure										
Racing or rough terrain cycling										
Cycling for pleasure										
Mowing the lawn — during the grass cutting season										
Watering the lawn or garden in the summer										
Digging, shovelling or chopping wood										
Weeding or pruning										
DIY e.g. carpentry, home or car maintenance										
High impact aerobics or step aerobics										
Other types of aerobics										
Exercises with weights										
Conditioning exercises e.g. using an exercise bike or rowing machine										
Floor exercises e.g. stretching, bending, keep fit or yoga										
Dancing e.g. ballroom or disco										
Competitive running										
Jogging										
Bowling — indoor, lawn or 10 pin										

	Number of times you did the activity in the last 12 months								Average time per episode	
	None	Less than once a month	Once a month	2 to 3 times a month	Once a week	2 to 3 times a week	4 to 5 times a week	Daily	Hours	Mins
Tennis or badminton										
Squash										
Table tennis										
Golf										
Football, rugby or hockey (during the season)										
Cricket (during the season)										
Rowing										
Netball, volleyball or basketball										
Fishing										
Horse-riding										
Snooker, billiards or darts										
Musical instrument playing or singing										
Ice-skating										
Sailing, wind-surfing or boating										
Martial arts, boxing or wrestling										

You have finished the questionnaire – *Thank you*

Your interviewer will collect it when he/she calls

v1.2,17 May 2010, ref: 10/H0308/50

Cam-CAN Self completion questionnaire

The Cambridge Centre for Ageing and Neuroscience Study (Cam-CAN)

Understanding healthy ageing: Lifespan development of brain and mind

Cam-CAN ID Number:

Date questionnaire completed:

This Cam-CAN paper questionnaire is intended to capture aspects of your life that are difficult to measure during the face-to face interview. It consists of two areas:

i) a lifetime experiences questionnaire
ii) a questionnaire on physical activity.

Please complete the questionnaire before your home interview; if you have any questions feel free to ring for advice.

The lifetime experiences captures two time periods in your life.

The two lifetime ages are

1. *Experiences from age 13 to age 29 (Young adulthood)*
2. *Experiences from age 30 to date (Midlife)*

To complete the questionnaire, please continue and answer each question as accurately as possible. Completing the questionnaire will take about 30-45 minutes. The Cam-CAN research interviewer will collect the questionnaire during the home interview.

Thank you for your participation.

LIFETIME EXPERIENCE QUESTIONNAIRE

This questionnaire covers your current activities, your training courses and your employment history. Please complete all sections relevant to you. You may be asked to skip sections.

YOUNG ADULTHOOD TRAINING

The following questions apply to the time in your life between 13 and 29 years of age.

Please detail all types of training or study undertaken from age 13 to 29.

Type of Course	Number of years completed	Full or part time
CSE		
NVQ level 1 / BTEC Introductory		
O level / GCSE / leaving certificate		
NVQ level 2 / BTEC First diploma		
A level / International baccalaureate		
NVQ level 3 / BTEC Diploma		
HNC / HND / NVQ level 4 / BTEC Professional		
BTEC Advanced		
College diploma		
University Undergraduate		
University Masters		
University PhD / Doctorate		
Clerical, administrative or book-keeping course		
Business course		
Trade apprenticeship		
Other professional course (specify)		
Other technical course (specify)		
Other graduate course (specify)		
Any other course (specify)		

YOUNG ADULTHOOD ACTIVITIES

Did you travel to any of the following places between the ages of 13 and 29?
Please tick (✓) all that apply

- ○ Around the UK/Ireland away from where I lived
- ○ Northern Europe / Scandinavia (e.g. France, Germany, Norway, Greenland)
- ○ Southern Europe (e.g. Italy, Spain)
- ○ Eastern Europe (e.g. Russia, Romania)
- ○ Northern Africa / Middle East (e.g. Tunisia, Egypt)
- ○ Southern Africa / Asia (e.g. Kenya, Madagascar, Mauritius, China, India)
- ○ North America (e.g. USA, Canada including Alaska, Hawaii)
- ○ South America / Central America / Caribbean (e.g. Mexico, Cuba, Brazil)
- ○ Australia / New Zealand / Pacific Islands / Antarctica (e.g. Fiji, Togo)
- ○ None of the above

From the ages of 13 to 29 please indicate how often you ever did the following activities (please tick ✓ box)

**Type of activity**	**Never**	**Less than once a month**	**Once a month**	**Every two weeks**	**Every week**	**Daily**
Make an outing to see a family member, friend or group of friends						
Practise or play a musical instrument						
Practise or develop an artistic pastime (e.g. drawing, acting, writing etc.)						
Mildly energetic activities (e.g. walking, carpentry, gardening, housework)						
Moderately energetic activities (e.g. dancing, golf, lawn mowing, easy cycling)						
Vigorous energetic activities (e.g. running, squash, competitive tennis)						
Read (material or any sort)						
Speak a second language						
Computer games / games consoles						
Social networking / internet surfing						
Crossword puzzles / sudoku						
Strategic games (e.g. chess)						
Prayer / religious activities						

MIDLIFE TRAINING

The following questions apply to the time in your life from the age of 30 to date.

Please detail all types of training or study undertaken from age 30 to date.

Type of Course	Number of years completed	Full or part time
CSE		
NVQ level 1 / BTEC Introductory		
O level / GCSE / leaving certificate		
NVQ level 2 / BTEC First diploma		
A level / International baccalaureate		
NVQ level 3 / BTEC Diploma		
HNC / HND / NVQ level 4 / BTEC Professional		
BTEC Advanced		
College diploma		
University Undergraduate		
University Masters		
University PhD / Doctorate		
Clerical, administrative or book-keeping course		
Business course		
Trade apprenticeship		
Other professional course (specify)		
Other technical course (specify)		
Other graduate course (specify)		
Any other course (specify)		

MIDLIFE ACTIVITIES

Have you travelled to any of the following places from the age of 30 to date?
Please tick (✓) all that apply

- ○ Around the UK/Ireland away from where I lived
- ○ Northern Europe / Scandinavia (e.g. France, Germany, Norway, Greenland)
- ○ Southern Europe (e.g. Italy, Spain)
- ○ Eastern Europe (e.g. Russia, Romania)
- ○ Northern Africa / Middle East (e.g. Tunisia, Egypt)
- ○ Southern Africa / Asia (e.g. Kenya, Madagascar, Mauritius, China, India)
- ○ North America (e.g. USA, Canada including Alaska, Hawaii)
- ○ South America / Central America / Caribbean (e.g. Mexico, Cuba, Brazil)
- ○ Australia / New Zealand / Pacific Islands / Antarctica (e.g. Fiji, Togo)
- ○ None of the above

From the ages of 30 to date please indicate how often you ever have done the following activities (please tick ✓ box)

**Type of activity**	**Never**	**Less than once a month**	**Once a month**	**Every two weeks**	**Every week**	**Daily**
Make an outing to see a family member, friend or group of friends						
Practise or play a musical instrument						
Practise or develop an artistic pastime (e.g. drawing, acting, writing etc.)						
Mildly energetic activities (e.g. walking, carpentry, gardening, housework)						
Moderately energetic activities (e.g. dancing, golf, lawn mowing, easy cycling)						
Vigorous energetic activities (e.g. running, squash, competitive tennis)						
Read (material or any sort)						
Speak a second language						
Computer games / games consoles						
Social networking / internet surfing						
Crossword puzzles / sudoku						
Strategic games (e.g. chess)						
Prayer / religious activities						

RECENT ACTIVITIES

What activities do you currently undertake during a typical week?
Please tick (✓) all that apply

- ○ Socialising (face-to-face)
- ○ Reading
- ○ Writing
- ○ Studying
- ○ Teaching
- ○ Volunteer work
- ○ Paid work
- ○ Social networking sites
- ○ Twitter / texting
- ○ None of the above
- ○ Prayer/ religious activity
- ○ Helping friends / family
- ○ Artistry
- ○ Strategic games (e.g. chess)
- ○ Learning something new
- ○ Hobby pastime
- ○ Brain training games
- ○ Other computer based games
- ○ Crossword puzzles / sudoku

What type of events or entertainment have you undertaken in the **last two months**? Please tick (✓) all that apply

- ○ Going to the cinema
- ○ Going to the pub
- ○ Going to pop concerts
- ○ Going to sporting events
- ○ None of the above
- ○ Going to the theatre
- ○ Going to a restaurant
- ○ Going to festivals
- ○ Going to clubs / societies

How do you usually acquire information about world and national events?
Please tick (✓) all that apply

- ○ No particular way
- ○ TV
- ○ Newspapers
- ○ Internet
- ○ Friends / word of mouth
- ○ Radio
- ○ Magazines
- ○ Other (please give details)

What kinds of materials are you reading on a regular basis?
Please tick (✓) all that apply

- ○ Newspaper articles
- ○ Novel / books
- ○ Other (please give details)
- ○ Magazine articles
- ○ Internet blogs / articles
- ○ None of the above

Have you travelled to any of the following places in the last **five** years?
Please tick (✓) all that apply

- ○ Around the UK/Ireland away from where I lived
- ○ Southern Europe (e.g. Italy, Spain)
- ○ Northern Africa / Middle East (e.g. Tunisia, Egypt)
- ○ North America (e.g. USA, Canada including Alaska, Hawaii)
- ○ Australia / New Zealand / Pacific Islands / Antarctica (e.g. Fiji, Togo)
- ○ Northern Europe / Scandinavia (e.g. France, Germany, Norway, Greenland)
- ○ Eastern Europe (e.g. Russia, Romania)
- ○ Southern Africa / Asia (e.g. Kenya, Madagascar, Mauritius, China, India)
- ○ South America / Central America / Caribbean (e.g. Mexico, Cuba, Brazil)
- ○ None of the above

BLANK PAGE

EMPLOYMENT

You will be asked about the **main jobs** that you have undertaken for different periods of your life. The enclosed guide gives examples of job titles and how we will code them. Please enter this sort of title. We also ask for the number of people you employed, or supervised during that period.

Example

**Age**	**SOC number (1-10) or JOB TITLE**	**Number of people in charge of**
18-19	Not employed	
20-24	Shop assistant	0
25-29	Secretary	0
30-34	Legal secretary	2
35-39	Personnel assistant	2
40-44	Human resources manager	>10
45-49	Human resources manager	>10
50-54	Human resources manager	>10
55-59	Human resources manager	4
60-64	Retired	0
65-69	Ages not applicable	
70-74	Ages not applicable	
75 years and above	Ages not applicable	

Please enter the job titles below, please try and be as explicit as possible. If you were in charge of people please indicate the approximate number. If you are not sure of the code enter your job title in the box provided.

**Age**	**SOC number (1-10) or JOB TITLE**	**Number of people in charge of**
18-19		
20-24		
25-29		
30-34		
35-39		
40-44		
45-49		
50-54		
55-59		
60-64		
65-69	Ages not applicable	
70-74	Ages not applicable	
75 years and above	Ages not applicable	

BLANK PAGE

PHYSICAL ACTIVITY

This questionnaire is designed to find out about your physical activity in your everyday life.

Please try to answer every question, except when there is a specific request to skip a section.

THE QUESTIONNAIRE IS DIVIDED INTO 3 SECTIONS

Section A asks about your physical activity patterns in and around the house.
Section B is about travel to work and your activity at work.
It may be skipped by people who have not worked at any stage during the last 12 months.
Section C asks about recreations in which you may have engaged during the last 12 months.

Section A HOME ACTIVITIES

GETTING UP AND GOING TO BED

Please put a time in **each** box

	Average over the past year	
	At what time do you normally get up?	At what time do you normally go to bed?
On a weekday		
On a weekend day		

GETTING ABOUT – Apart from going to work

Which form of transport do you use **most often** apart from your journey to and from work?

Please tick (✓) one box **ONLY** per line

Distance of journeys	Usual mode of transport			
	Car	Walk	Public transport	Cycle
Less than one mile				
1–5 mile(s)				
More than 5 miles				

TV OR VIDEO VIEWING

Please put a tick (✓) on **EVERY** line

Hours of TV or Video watched per day	Average over the last 12 months					
	None	Less than 1	1 to 2	2 to 3	3 to 4	More than 4
On a weekday before 6 pm						
On a weekday after 6 pm						
On a weekend day before 6 pm						
On a weekend day after 6 pm						

STAIR CLIMBING AT HOME

Please put a tick (✓) on **EVERY** line

Number of times you climbed up a flight of stairs (approx 10 steps) each day at home	**Average over the last 12 months**					
	None	1 to 5	6 to 10	11 to 15	16 to 20	More than 20
On a weekday						
On a weekend day						

ACTIVITIES IN AND AROUND THE HOME

Please put a tick (✓) on **EVERY** line

Approximate number of hours each week	**Average hours per week over the last 12 months**						
	0	Less than 1	1 to 3	3 to 6	6 to 10	10 to 15	>15
Preparing food, cooking and washing up							
Shopping for food and groceries							
Shopping and browsing in shops for other items (e.g. clothes, toys)							
Cleaning the house							
Doing the laundry and ironing							
Caring for pre-school children or babies at home (not as paid employment)							
Caring for handicapped, elderly or disabled people at home (not as paid employment)							

Section B ACTIVITY AT WORK

Please answer this section **only** if you have been in paid employment at any time during the last 12 months or you have done regular, organised voluntary work.

IF YOU HAVE NOT BEEN EMPLOYED DURING THE LAST 12 MONTHS PLEASE GO TO PAGE 18

TYPES OF WORK DURING THE LAST TWELVE MONTHS

- We would like to know what full or part-time jobs you have done in the last 12 months.
- You may have held a single job or have held two jobs at once.
- If you have changed jobs with the same employer, you should enter it as a change of job **only** if it entailed a substantial change in physical effort.

EXAMPLE

Someone who worked full-time for 6 months, then retired, rested for 3 months and then started a voluntary job for 6 hours a week, would complete the questions as follows

	**Job 1**	**Job 2**
Name of occupation	nurse	shop work
How many hours **per week** did you usually work?	38	6
For how many months in the last 12 months did you do this work?	6	3

Example

ACTIVITY LEVELS AT YOUR WORK
Please complete EACH line

	**Job 1**			**Job 2**		
	No	Yes	Hours per week	No	Yes	Hours per week
Sitting — light work<br>e.g. desk work, or driving a car or truck		✓	6	✓		
Sitting — moderate work<br>e.g. working heavy levers or riding a mower or forklift truck	✓				✓	2
Standing — light work<br>e.g. lab technician work or working at a shop counter		✓	30		✓	4
Standing — light/moderate work<br>e.g. light welding or stocking shelves		✓	2	✓		

Example

The number of hours in each activity should add up to the number of hours that you worked in each job e.g. 6+30+2=38 (nurse)

What jobs have you held in the last 12 months, and how many months in the year did you do them?

Please complete EACH line

	Job 1	Job 2
Name of occupation		
How many hours **per week** did you usually work?		
For how many months in the last 12 months did you do this work?		

ACTIVITY LEVELS AT YOUR WORK

Now we would like you to take the total number of hours you worked per week in each job and divide them up according to your activity level.

Please complete **EACH** line

	Job 1			Job 2		
	No	Yes	Hours per week	No	Yes	Hours per week
Sitting — light work<br>e.g. desk work, or driving a car or truck						
Sitting — moderate work<br>e.g. working heavy levers or riding a mower or forklift truck						
Standing — light work<br>e.g. lab technician work or working at a shop counter						
Standing — light/moderate work e.g. light welding or stocking shelves						
Standing — moderate work<br>e.g. fast rate assembly line work or lifting up to 50 lbs (25 kg) every 5 minutes for a few seconds at a time						
Standing — moderate/heavy work<br>e.g. masonry/painting or lifting more than 50 lbs (25 kg) every 5 minutes for a few seconds at a time						
Walking at work — carrying nothing heavier than a briefcase e.g. moving about a shop						
Walking — carrying something heavy						
Moving, pushing heavy objects weighing over 75lbs (35kg)						

STAIR OR STEP CLIMBING AT WORK

Please put a tick (✓) on EACH line where appropriate

Number of times a day you climbed up a flight of stairs (10 steps) at work	AVERAGE NUMBER OF TIMES A DAY OVER THE LAST 12 MONTHS					
	0	1 to 5	6 to 10	11 to 15	16 to 20	More than 20
Job 1						
Job 2						

Number of times a day you climbed up a ladder at work	AVERAGE NUMBER OF TIMES A DAY OVER THE LAST 12 MONTHS					
	0	1 to 5	6 to 10	11 to 15	16 to 20	More than 20
Job 1						
Job 2						

In an average working day in **job 1** did you (please tick ✓ all that apply)

- ○ Kneel for more than an hour in total?
- ○ Squat for more than an hour in total?
- ○ Get up from kneeling or squatting more than 30 times?

In an average working day in **job 2** did you

- ○ Kneel for more than an hour in total?
- ○ Squat for more than an hour in total?
- ○ Get up from kneeling or squatting more than 30 times?

TRAVEL TO AND FROM WORK

JOB 1
Please complete **EVERY** line

Roughly how many miles was it from home to Job 1?	
How many times a week did you travel from home to Job 1?	

Please tick (✓) one box **ONLY** per line

How did you normally travel to Job 1?	Always	Usually	Occasionally	Never or rarely
By car				
By works or public transport				
By bicycle				
Walking				

JOB 2 *(if appropriate)*
Please complete **EVERY** line

Roughly how many miles was it from home to Job 2?	
How many times a week did you travel from home to Job 2?	

Please tick (✓) one box **ONLY** per line

How did you normally travel to Job 2?	Always	Usually	Occasionally	Never or rarely
By car				
By works or public transport				
By bicycle				
Walking				

Section C RECREATION

The following questions ask about how you spent your leisure time. Please indicate how often you did each activity on average over the last 12 months. For activities that are seasonal, e.g. cricket or mowing the lawn, please put the average frequency during the season when you did the activity. Please indicate the average length of time that you spent doing the activity on each occasion.

EXAMPLE
If you had mowed the lawn every fortnight in the grass cutting season and took 1 hour and 10 minutes on each occasion, and you went walking for pleasure for 40 minutes once a week, you would complete the table below as follows:

Please give an answer for the AVERAGE TIME you spent on each activity and the NUMBER OF TIMES you did that activity in the past year.

	Number of times you did the activity in the last 12 months								Average time per episode	
	None	Less than once a month	Once a month	2 to 3 times a month	Once a week	2 to 3 times a week	4 to 5 times a week	Daily	Hours	Mins
Mowing the lawn				✓					1	10
Walking for pleasure					✓					40

Example

For activities you do as a means of transportation please do not included those in this table.

Now please complete the table on pages 19-20

Please give an answer for the NUMBER OF TIMES you did the following activities in the last 12 months and the AVERAGE TIME you spent on each activity.

Please complete EACH line

	Number of times you did the activity in the last 12 months								Average time per episode	
	None	Less than once a month	Once a month	2 to 3 times a month	Once a week	2 to 3 times a week	4 to 5 times a week	Daily	Hours	Mins
Swimming — competitive										
Swimming — leisurely										
Backpacking or mountain climbing										
Walking for pleasure										
Racing or rough terrain cycling										
Cycling for pleasure										
Mowing the lawn — during the grass cutting season										
Watering the lawn or garden in the summer										
Digging, shovelling or chopping wood										
Weeding or pruning										
DIY e.g. carpentry, home or car maintenance										
High impact aerobics or step aerobics										
Other types of aerobics										
Exercises with weights										
Conditioning exercises e.g. using an exercise bike or rowing machine										
Floor exercises e.g. stretching, bending, keep fit or yoga										
Dancing e.g. ballroom or disco										
Competitive running										
Jogging										
Bowling — indoor, lawn or 10 pin										

	Number of times you did the activity in the last 12 months								Average time per episode	
	None	Less than once a month	Once a month	2 to 3 times a month	Once a week	2 to 3 times a week	4 to 5 times a week	Daily	Hours	Mins
Tennis or badminton										
Squash										
Table tennis										
Golf										
Football, rugby or hockey (during the season)										
Cricket (during the season)										
Rowing										
Netball, volleyball or basketball										
Fishing										
Horse-riding										
Snooker, billiards or darts										
Musical instrument playing or singing										
Ice-skating										
Sailing, wind-surfing or boating										
Martial arts, boxing or wrestling										

You have finished the questionnaire – *Thank you*

Your interviewer will collect it when he/she calls

Cam-CAN Self completion questionnaire

The Cambridge Centre for Ageing and Neuroscience Study (Cam-CAN)

Understanding healthy ageing: Lifespan development of brain and mind

Cam-CAN ID Number:

Date questionnaire completed:

This Cam-CAN paper questionnaire is intended to capture aspects of your life that are difficult to measure during the face-to face interview. It consists of two areas:

i) a lifetime experiences questionnaire
ii) a questionnaire on physical activity.

Please complete the questionnaire before your home interview; if you have any questions feel free to ring for advice.

The lifetime experiences captures three time periods in your life.

The three lifetime ages are

1. *Experiences from age 13 to age 29 (Young adulthood)*
2. *Experiences from age 30 to age 64 (Midlife)*
3. *Experiences since the age of 65 (Later life)*

To complete the questionnaire, please continue and answer each question as accurately as possible. Completing the questionnaire will take about 40-60 minutes. The Cam-CAN research interviewer will collect the questionnaire during the home interview.

Thank you for your participation.

LIFETIME EXPERIENCE QUESTIONNAIRE

This questionnaire covers your current activities, your training courses and your employment history. Please complete all sections relevant to you. You may be asked to skip sections.

YOUNG ADULTHOOD TRAINING

The following questions apply to the time in your life between 13 and 29 years of age.

Please detail all types of training or study undertaken from age 13 to 29.

**Type of Course**	**Number of years completed**	**Full or part time**
CSE		
NVQ level 1 / BTEC Introductory		
O level / GCSE / leaving certificate		
NVQ level 2 / BTEC First diploma		
A level / International baccalaureate		
NVQ level 3 / BTEC Diploma		
HNC / HND / NVQ level 4 / BTEC Professional		
BTEC Advanced		
College diploma		
University Undergraduate		
University Masters		
University PhD / Doctorate		
Clerical, administrative or book-keeping course		
Business course		
Trade apprenticeship		
Other professional course<br>(specify)		
Other technical course<br>(specify)		
Other graduate course<br>(specify)		
Any other course<br>(specify)		

YOUNG ADULTHOOD ACTIVITIES

Did you travel to any of the following places between the ages of 13 and 29?
Please tick (✓) all that apply

- ○ Around the UK/Ireland away from where I lived
- ○ Northern Europe / Scandinavia (e.g. France, Germany, Norway, Greenland)
- ○ Southern Europe (e.g. Italy, Spain)
- ○ Eastern Europe (e.g. Russia, Romania)
- ○ Northern Africa / Middle East (e.g. Tunisia, Egypt)
- ○ Southern Africa / Asia (e.g. Kenya, Madagascar, Mauritius, China, India)
- ○ North America (e.g. USA, Canada including Alaska, Hawaii)
- ○ South America / Central America / Caribbean (e.g. Mexico, Cuba, Brazil)
- ○ Australia / New Zealand / Pacific Islands / Antarctica (e.g. Fiji, Togo)
- ○ None of the above

From the ages of 13 to 29 please indicate how often you ever did the following activities (please tick ✓ box)

**Type of activity**	**Never**	**Less than once a month**	**Once a month**	**Every two weeks**	**Every week**	**Daily**
Make an outing to see a family member, friend or group of friends						
Practise or play a musical instrument						
Practise or develop an artistic pastime (e.g. drawing, acting, writing etc.)						
Mildly energetic activities (e.g. walking, carpentry, gardening, housework)						
Moderately energetic activities (e.g. dancing, golf, lawn mowing, easy cycling)						
Vigorous energetic activities (e.g. running, squash, competitive tennis)						
Read (material or any sort)						
Speak a second language						
Computer games / games consoles						
Social networking / internet surfing						
Crossword puzzles / sudoku						
Strategic games (e.g. chess)						
Prayer /religious activities						

MIDLIFE TRAINING

The following questions apply to the time in your life between 30 and 64 years of age.

Please detail all types of training or study undertaken from age 30 and 64.

Type of Course	Number of years completed	Full or part time
CSE		
NVQ level 1 / BTEC Introductory		
O level / GCSE / leaving certificate		
NVQ level 2 / BTEC First diploma		
A level / International baccalaureate		
NVQ level 3 / BTEC Diploma		
HNC / HND / NVQ level 4 / BTEC Professional		
BTEC Advanced		
College diploma		
University Undergraduate		
University Masters		
University PhD / Doctorate		
Clerical, administrative or book-keeping course		
Business course		
Trade apprenticeship		
Other professional course (specify)		
Other technical course (specify)		
Other graduate course (specify)		
Any other course (specify)		

MIDLIFE ACTIVITIES

Between the ages of 30 and 64 did you ever travel to any of the following places? Please tick (✓) all that apply

- ○ Around the UK/Ireland away from where I lived
- ○ Northern Europe / Scandinavia (e.g. France, Germany, Norway, Greenland)
- ○ Southern Europe (e.g. Italy, Spain)
- ○ Eastern Europe (e.g. Russia, Romania)
- ○ Northern Africa / Middle East (e.g. Tunisia, Egypt)
- ○ Southern Africa / Asia (e.g. Kenya, Madagascar, Mauritius, China, India)
- ○ North America (e.g. USA, Canada including Alaska, Hawaii)
- ○ South America / Central America / Caribbean (e.g. Mexico, Cuba, Brazil)
- ○ Australia / New Zealand / Pacific Islands / Antarctica (e.g. Fiji, Togo)
- ○ None of the above

From the ages of 30 to 64 please indicate how often you ever did the following activities (please tick ✓ box)

**Type of activity**	**Never**	**Less than once a month**	**Once a month**	**Every two weeks**	**Every week**	**Daily**
Make an outing to see a family member, friend or group of friends						
Practise or play a musical instrument						
Practise or develop an artistic pastime (e.g. drawing, acting, writing etc.)						
Mildly energetic activities (e.g. walking, carpentry, gardening, housework)						
Moderately energetic activities (e.g. dancing, golf, lawn mowing, easy cycling)						
Vigorous energetic activities (e.g. running, squash, competitive tennis)						
Read (material or any sort)						
Speak a second language						
Computer games / games consoles						
Social networking / internet surfing						
Crossword puzzles / sudoku						
Strategic games (e.g. chess)						
Prayer / religious activities						

LATER LIFE TRAINING

The following questions apply to the time in your life from the age of 65.

Please detail all types of training or study undertaken from age 65 to date.

Type of Course	Number of years completed	Full or part time
CSE		
NVQ level 1 / BTEC Introductory		
O level / GCSE / leaving certificate		
NVQ level 2 / BTEC First diploma		
A level / International baccalaureate		
NVQ level 3 / BTEC Diploma		
HNC / HND / NVQ level 4 / BTEC Professional		
BTEC Advanced		
College diploma		
University Undergraduate		
University Masters		
University PhD / Doctorate		
Clerical, administrative or book-keeping course		
Business course		
Trade apprenticeship		
Other professional course (specify)		
Other technical course (specify)		
Other graduate course (specify)		
Any other course (specify)		

LATER LIFE ACTIVITIES

The following questions apply to the time in your life from 65 years of age to date

Have you ever travelled to any of the following places from the age of 65 to date?
Please tick (✓) all that apply

- ○ Around the UK/Ireland away from where I lived
- ○ Northern Europe / Scandinavia (e.g. France, Germany, Norway, Greenland)
- ○ Southern Europe (e.g. Italy, Spain)
- ○ Eastern Europe (e.g. Russia, Romania)
- ○ Northern Africa / Middle East (e.g. Tunisia, Egypt)
- ○ Southern Africa / Asia (e.g. Kenya, Madagascar, Mauritius, China, India)
- ○ North America (e.g. USA, Canada including Alaska, Hawaii)
- ○ South America / Central America / Caribbean (e.g. Mexico, Cuba, Brazil)
- ○ Australia / New Zealand / Pacific Islands / Antarctica (e.g. Fiji, Togo)
- ○ None of the above

How frequently would you say you have ever taken part in the following activities since the age of 65 (please tick ✓ box)

Type of activity	Never	Less than once a month	Once a month	Every two weeks	Every week	Daily
Make an outing to see a family member, friend or group of friends						
Practise or play a musical instrument						
Practise or develop an artistic pastime (e.g. drawing, acting, writing etc.)						
Mildly energetic activities (e.g. walking, carpentry, gardening, housework)						
Moderately energetic activities (e.g. dancing, golf, lawn mowing, easy cycling)						
Vigorous energetic activities (e.g. running, squash, competitive tennis)						
Read (material of any sort)						
Speak a second language						
Computer games / games consoles						
Social networking / internet surfing						
Crossword puzzles / sudoku						
Strategic games (e.g. chess)						
Prayer / religious activities						

RECENT ACTIVITIES

What activities do you currently undertake during a typical week?
Please tick (✓) all that apply

- ○ Socialising (face-to-face)
- ○ Reading
- ○ Writing
- ○ Studying
- ○ Teaching
- ○ Volunteer work
- ○ Paid work
- ○ Social networking sites
- ○ Twitter / texting
- ○ None of the above
- ○ Prayer / religious activity
- ○ Helping friends / family
- ○ Artistry
- ○ Strategic games (e.g. chess)
- ○ Learning something new
- ○ Hobby pastime
- ○ Brain training games
- ○ Other computer based games
- ○ Crossword puzzles / sudoku

What type of events or entertainment have you undertaken in the **last two months**? Please tick (✓) all that apply

- ○ Going to the cinema
- ○ Going to the pub
- ○ Going to pop concerts
- ○ Going to sporting events
- ○ None of the above
- ○ Going to the theatre
- ○ Going to a restaurant
- ○ Going to festivals
- ○ Going to clubs / societies

How do you usually acquire information about world and national events?
Please tick (✓) all that apply

- ○ No particular way
- ○ TV
- ○ Newspapers
- ○ Internet
- ○ Friends / word of mouth
- ○ Radio
- ○ Magazines
- ○ Other (please give details)

What kinds of materials are you reading on a regular basis?
Please tick (✓) all that apply

- ○ Newspaper articles
- ○ Novels / books
- ○ Other (please give details)
- ○ Magazine articles
- ○ Internet blogs / articles
- ○ None of the above

Have you travelled to any of the following places in the last **five** years?
Please tick (✓) all that apply

- ○ Around the UK/Ireland away from where I lived
- ○ Southern Europe (e.g. Italy, Spain)
- ○ Northern Africa / Middle East (e.g. Tunisia, Egypt)
- ○ North America (e.g. USA, Canada including Alaska, Hawaii)
- ○ Australia / New Zealand / Pacific Islands / Antarctica (e.g. Fiji, Togo)
- ○ Northern Europe / Scandinavia (e.g. France, Germany, Norway, Greenland)
- ○ Eastern Europe (e.g. Russia, Romania)
- ○ Southern Africa / Asia (e.g. Kenya, Madagascar, Mauritius, China, India)
- ○ South America / Central America / Caribbean (e.g. Mexico, Cuba, Brazil)
- ○ None of the above

BLANK PAGE

EMPLOYMENT

You will be asked about the **main jobs** that you have undertaken for different periods of your life. The enclosed guide gives examples of job titles and how we will code them. Please enter this sort of title. We also ask for the number of people you employed, or supervised during that period.

Example

Age	SOC number (1-10) or JOB TITLE	Number of people in charge of
18-19	Not employed	
20-24	Shop assistant	0
25-29	Secretary	0
30-34	Legal secretary	2
35-39	Personnel assistant	2
40-44	Human resources manager	>10
45-49	Human resources manager	>10
50-54	Human resources manager	>10
55-59	Human resources manager	>10
60-64	Human resources manager	>10
65-69	Retired	0
70-74		
75 years and above		

Please enter the job titles below, please try and be as explicit as possible. If you were in charge of people please indicate the approximate number. If you are not sure of the code enter your job title in the box provided.

**Age**	**SOC number (1-10) or JOB TITLE**	**Number of people in charge of**
18-19		
20-24		
25-29		
30-34		
35-39		
40-44		
45-49		
50-54		
55-59		
60-64		
65-69		
70-74		
75 years and above		

BLANK PAGE

PHYSICAL ACTIVITY

This questionnaire is designed to find out about your physical activity in your everyday life.

Please try to answer every question, except when there is a specific request to skip a section.

THE QUESTIONNAIRE IS DIVIDED INTO 3 SECTIONS

Section A asks about your physical activity patterns in and around the house.
Section B is about travel to work and your activity at work.
It may be skipped by people who have not worked at any stage during the last 12 months.
Section C asks about recreations in which you may have engaged during the last 12 months.

Section A HOME ACTIVITIES

GETTING UP AND GOING TO BED

Please put a time in **each** box

	Average over the past year	
	At what time do you normally get up?	At what time do you normally go to bed?
On a weekday		
On a weekend day		

GETTING ABOUT – Apart from going to work

Which form of transport do you use **most often** apart from your journey to and from work?

Please tick (✓) one box **ONLY** per line

Distance of journeys	Usual mode of transport			
	Car	Walk	Public transport	Cycle
Less than one mile				
1–5 mile(s)				
More than 5 miles				

TV OR VIDEO VIEWING

Please put a tick (✓) on **EVERY** line

Hours of TV or Video watched per day	Average over the last 12 months					
	None	Less than 1	1 to 2	2 to 3	3 to 4	More than 4
On a weekday before 6 pm						
On a weekday after 6 pm						
On a weekend day before 6 pm						
On a weekend day after 6 pm						

STAIR CLIMBING AT HOME

Please put a tick (✓) on **EVERY** line

Number of times you climbed up a flight of stairs (approx 10 steps) each day at home	**Average over the last 12 months**					
	None	1 to 5	6 to 10	11 to 15	16 to 20	More than 20
On a weekday						
On a weekend day						

ACTIVITIES IN AND AROUND THE HOME

Please put a tick (✓) on **EVERY** line

Approximate number of hours each week	**Average hours per week over the last 12 months**						
	0	Less than 1	1 to 3	3 to 6	6 to 10	10 to 15	>15
Preparing food, cooking and washing up							
Shopping for food and groceries							
Shopping and browsing in shops for other items (e.g. clothes, toys)							
Cleaning the house							
Doing the laundry and ironing							
Caring for pre-school children or babies at home (not as paid employment)							
Caring for handicapped, elderly or disabled people at home (not as paid employment)							

Section B ACTIVITY AT WORK

Please answer this section **only** if you have been in paid employment at any time during the last 12 months or you have done regular, organised voluntary work.

IF YOU HAVE NOT BEEN EMPLOYED DURING THE LAST 12 MONTHS PLEASE GO TO PAGE 20

TYPES OF WORK DURING THE LAST TWELVE MONTHS

- We would like to know what full or part-time jobs you have done in the last 12 months.
- You may have held a single job or have held two jobs at once.
- If you have changed jobs with the same employer, you should enter it as a change of job **only** if it entailed a substantial change in physical effort.

EXAMPLE

Someone who worked full-time for 6 months, then retired, rested for 3 months and then started a voluntary job for 6 hours a week, would complete the questions as follows

	**Job 1**	**Job 2**
Name of occupation	nurse	shop work
How many hours **per week** did you usually work?	38	6
For how many months in the last 12 months did you do this work?	6	3

Example

ACTIVITY LEVELS AT YOUR WORK

Please complete EACH line

	**Job 1**			**Job 2**		
	No	Yes	Hours per week	No	Yes	Hours per week
Sitting — light work<br>e.g. desk work, or driving a car or truck		✓	6	✓		
Sitting — moderate work<br>e.g. working heavy levers or [illegible]ling a mower or forklift truck	✓				✓	2
Standing — light work<br>e.g. lab technician work or working at a shop counter		✓	30		✓	4
Standing — light/moderate work<br>e.g. light welding or stocking shelves		✓	2	✓		

Example

The number of hours in each activity should add up to the number of hours that you worked in each job e.g. 6+30+2=38 (nurse)

What jobs have you held in the last 12 months, and how many months in the year did you do them?

Please complete EACH line

	**Job 1**	**Job 2**
Name of occupation		
How many hours **per week** did you usually work?		
For how many months in the last 12 months did you do this work?		

ACTIVITY LEVELS AT YOUR WORK

Now we would like you to take the total number of hours you worked per week in each job and divide them up according to your activity level.

Please complete **EACH** line

	**Job 1**			**Job 2**		
	No	Yes	Hours per week	No	Yes	Hours per week
Sitting — light work e.g. desk work, or driving a car or truck						
Sitting — moderate work e.g. working heavy levers or riding a mower or forklift truck						
Standing — light work e.g. lab technician work or working at a shop counter						
Standing — light/moderate work e.g. light welding or stocking shelves						
Standing — moderate work e.g. fast rate assembly line work or lifting up to 50 lbs (25 kg) every 5 minutes for a few seconds at a time						
Standing — moderate/heavy work e.g. masonry/painting or lifting more than 50 lbs (25 kg) every 5 minutes for a few seconds at a time						
Walking at work — carrying nothing heavier than a briefcase e.g. moving about a shop						
Walking — carrying something heavy						
Moving, pushing heavy objects weighing over 75lbs (35kg)						

STAIR OR STEP CLIMBING AT WORK

Please put a tick (✓) on EACH line where appropriate

Number of times a day you climbed up a flight of stairs (10 steps) at work	AVERAGE NUMBER OF TIMES A DAY OVER THE LAST 12 MONTHS					
	0	1 to 5	6 to 10	11 to 15	16 to 20	More than 20
Job 1						
Job 2						

Number of times a day you climbed up a ladder at work	AVERAGE OVER THE LAST 12 MONTHS					
	0	1 to 5	6 to 10	11 to 15	16 to 20	More than 20
Job 1						
Job 2						

In an average working day in **job 1** did you (please tick ✓ all that apply)

- ○ Kneel for more than an hour in total?
- ○ Squat for more than an hour in total?
- ○ Get up from kneeling or squatting more than 30 times?

In an average working day in **job 2** did you

- ○ Kneel for more than an hour in total?
- ○ Squat for more than an hour in total?
- ○ Get up from kneeling or squatting more than 30 times?

TRAVEL TO AND FROM WORK

JOB 1

Please complete **EVERY** line

Roughly how many miles was it from home to Job 1?	
How many times a week did you travel from home to Job 1?	

Please tick (✓) one box ONLY per line

How did you normally travel to Job 1?	Always	Usually	Occasionally	Never or rarely
By car				
By works or public transport				
By bicycle				
Walking				

JOB 2

Please complete **EVERY** line

Roughly how many miles was it from home to Job 2?	
How many times a week did you travel from home to Job 2?	

Please tick (✓) one box ONLY per line

How did you normally travel to Job 2?	Always	Usually	Occasionally	Never or rarely
By car				
By works or public transport				
By bicycle				
Walking				

Section C RECREATION

The following questions ask about how you spent your leisure time. Please indicate how often you did each activity on average over the last 12 months. For activities that are seasonal, e.g. cricket or mowing the lawn, please put the average frequency during the season when you did the activity. Please indicate the average length of time that you spent doing the activity on each occasion.

EXAMPLE
If you had mowed the lawn every fortnight in the grass cutting season and took 1 hour and 10 minutes on each occasion, and you went walking for pleasure for 40 minutes once a week, you would complete the table below as follows:

Please give an answer for the AVERAGE TIME you spent on each activity and the NUMBER OF TIMES you did that activity in the past year.

Example

	Number of times you did the activity in the last 12 months								Average time per episode	
	None	Less than once a month	Once a month	2 to 3 times a month	Once a week	2 to 3 times a week	4 to 5 times a week	Daily	Hours	Mins
Mowing the lawn				✓					1	10
Walking for pleasure					✓					40

For activities you do as a means of transportation please do not include those in this table.

Now please complete the table on pages 21-22

Please give an answer for the NUMBER OF TIMES you did the following activities in the last 12 months and the AVERAGE TIME you spent on each activity.

Please complete EACH line

	Number of times you did the activity in the last 12 months								Average time per episode	
	None	Less than once a month	Once a month	2 to 3 times a month	Once a week	2 to 3 times a week	4 to 5 times a week	Daily	Hours	Mins
Swimming — competitive										
Swimming — leisurely										
Backpacking or mountain climbing										
Walking for pleasure										
Racing or rough terrain cycling										
Cycling for pleasure										
Mowing the lawn — during the grass cutting season										
Watering the lawn or garden in the summer										
Digging, shovelling or chopping wood										
Weeding or pruning										
DIY e.g. carpentry, home or car maintenance										
High impact aerobics or step aerobics										
Other types of aerobics										
Exercises with weights										
Conditioning exercises e.g. using an exercise bike or rowing machine										
Floor exercises e.g. stretching, bending, keep fit or yoga										
Dancing e.g. ballroom or disco										
Competitive running										
Jogging										
Bowling — indoor, lawn or 10 pin										

	Number of times you did the activity in the last 12 months								Average time per episode	
	None	Less than once a month	Once a month	2 to 3 times a month	Once a week	2 to 3 times a week	4 to 5 times a week	Daily	Hours	Mins
Tennis or badminton										
Squash										
Table tennis										
Golf										
Football, rugby or hockey (during the season)										
Cricket (during the season)										
Rowing										
Netball, volleyball or basketball										
Fishing										
Horse-riding										
Snooker, billiards or darts										
Musical instrument playing or singing										
Ice-skating										
Sailing, wind-surfing or boating										
Martial arts, boxing or wrestling										

You have finished the questionnaire – *Thank you*

Your interviewer will collect it when he/she calls
